# Inpatient Flow Distribution Patterns at Shanghai Hospitals

**DOI:** 10.3390/ijerph17072183

**Published:** 2020-03-25

**Authors:** Xuechen Xiong, Li Luo

**Affiliations:** Collaborative Innovation Center of Health Risks Governance, School of Public Health, Fudan University, Shanghai 200433, China; 18111020029@fudan.edu.cn

**Keywords:** inpatient flow, distribution patterns, empirical study, Shanghai

## Abstract

Empirical studies based on patient flow data are needed to provide more materials to summarize the general pattern of patient distribution models. This study takes Shanghai as an example and tries to demonstrate the inpatient flow distribution model for different levels and specialties of medical institutions. Power, negative exponential, Gaussian, and log-logistic models were used to fit the distributions of inpatients, and a model of inpatient distribution patterns in Shanghai was derived, based on these four models. Then, the adjusted coefficient of determination (R^2^) and Akaike information criterion (AIC) values were used to assess the model fitting effect. The log-logistic function model has a good simulation effect and the strongest applicability in most hospitals. The estimated value of the distance-decay parameter β in the log-logistic function model is 1.67 for all patients, 1.89 for regional hospital inpatients, 1.40 for tertiary hospital inpatients, 1.64 for traditional Chinese medicine hospital inpatients, and 0.85 for mental hospital inpatients. However, the simulations at the tumor, children’s and maternity hospitals, were not satisfactory. Based on the results of empirical analysis, the four attenuation coefficient models are valid in Shanghai, and the log-logistic model of the inpatient distributions at most hospitals have good simulation effects. However, further in-depth analysis combined with the characteristics of specific specialties is needed to obtain the inpatient model in line with the characteristics of these specialties.

## 1. Background

### 1.1. Shanghai Medical Service System

As one of the largest cities in China and the medical center of eastern China, Shanghai has the highest level of medical technology in China, with relatively abundant medical resources. There are approximately 5.74 beds and 3.0 doctors for every thousand Shanghai residents [1]; both statistics are highly ranked among all the cities in China. In Shanghai, medical institutions are divided into three categories: primary medical institutions, regional medical institutions, and tertiary medical institutions. Primary medical institutions mainly provide basic medical services, which are limited to neighboring residents. Regional medical institutions mainly provide outpatient and inpatient services for common and frequently occurring diseases. Tertiary medical institutions mainly provide outpatient and inpatient services for difficult diseases and treat patients, including, but not limited to, Shanghai residents [2]. Most medical institutions, especially regional medical institutions and tertiary medical institutions, are public. The government has strict restrictions on the pricing of medical service. The loss of public hospitals are paid by the financial department of the government. All public medical institutions can enjoy medical insurance policy. Currently, a main problem of the medical service system in Shanghai is that the total amount of medical resources is relatively insufficient and the structure of medical resources utilization is unreasonable. In 2018, Shanghai’s total per capita health spending was about $1232. China’s total per capita health expenditure is about $688; in the USA it is $10,586, and Australia $5395 [3]. Compared with the United States and other western developed countries, the per capita health expenditure of Shanghai still lags behind, to some extent. From the composition of health expenses, in 2018, the total health expenditure in Shanghai reached $5.7 trillion dollars, of which the health expenses in hospitals accounted for 78% and the health expenses in primary medical institutions accounted for about 15%. Among the hospital health expenses, tertiary hospital account for 71%, secondary hospital account for 28%, and primary medical institutions account for about 1% [1]. At present, a new round of medical reform has been developed. The government currently advocates graded diagnosis and treatment [4], which means encouraging residents to choose appropriate medical institutions according to disease severity, community hospitals for minor diseases, and tertiary medical institutions for major diseases to improve the utilization efficiency of medical resources at all levels. Local hospitalization has been recommended by many countries because it reduces travel and medical cost for patients, balances the supply and demand of healthcare services, maintains local hospital revenue, and improves patient-doctor relationships overall [5]. The study on the flow of patients in this paper is helpful for understanding the medical behavior habits of the population and provides a reference for adjusting medical behavior patterns.

### 1.2. Quantitative Model for Descripting Patient Flow

The gravity model can be used to analyze and predict spatial flows. It holds that phenomena are interrelated and restricted in geographic space. According to the distance attenuation law, if geographical phenomena interact with each other, the action decreases with increasing distance. In the area of health, understanding patient travel behaviors for seeking hospital care is fundamental for analyzing the healthcare market and planning resource allocation [6]. However, patient flow analysis is complex and requires actual traffic flow data, which are difficult to obtain. The key parameters, such as the distance attenuation coefficient, usually vary from temporally and spatially. There are relatively few empirical studies on the gravity model for patient populations [7]. Therefore, empirical studies based on patient-flow data in increasingly more places are needed to provide more references for other similar areas, and more materials to summarize the general pattern of patient-distribution models [8,9]. In addition, some studies have begun to focus on the heterogeneity of medical services [10]. One study examined the differences in medical treatment patterns among different ages, genders, and ethnic groups and determined the specific distance decay parameter on hospital inpatient visits among subpopulations [6,11]. Factors, such as age and gender, are not direct factors influencing the choice of medical treatment. The direct reasons influencing the choice of medical treatment are disease type and severity, while factors, such as age and gender, are the reasons influencing disease type and severity [12]. Therefore, considering individual disease, it is more appropriate to study the differences in medical treatment patterns based on the classification of diseases. Considering disease severity, it is more appropriate to study differences in medical treatment patterns based on the institutional classification. This study takes Shanghai inpatients as an example and attempts to demonstrate the inpatient flow distribution model of different levels and specialties of medical institutions in Shanghai, as well as provide empirical results for reference.

## 2. Method

### 2.1. Data Sources and Processing

#### 2.1.1. Population Data

In 2016, Shanghai’s resident population was approximately 24 million people. The resident population of each residential building in Shanghai was calculated from the Public Security Department of Shanghai. By locating the residential buildings, the population distribution can be determined [13].

#### 2.1.2. Inpatient Flow Data

The data of inpatients in Shanghai were collected from the first page of the inpatient medical record from each medical institution in Shanghai in 2016. This page records the information of each inpatient, including the patient’s residential address, medical institution, major disease diagnoses, and treatment(s). In 2016, the total number of inpatients in all medical institutions in Shanghai was approximately 3 million people, including approximately 2.5 million local residents and 0.5 million non-local residents. The data were from the Shanghai Health Bureau. Considering the limitations of the study area, this paper only used the data of the local residents in Shanghai when calculating the distribution pattern of inpatient flow in Shanghai.

#### 2.1.3. Hospital Information

The information of medical institutions came from the Shanghai Health Bureau. In 2016, Shanghai had approximately 5629 medical institutions, including 5433 primary medical institutions, 160 regional medical institutions, and 36 tertiary medical institutions. Inpatient services were mainly provided by regional medical institutions and tertiary medical institutions. This study mainly focused on the medical institutions providing inpatient services, and then we collected these institutions’ characteristics, including the level, type, number of beds, etc. In the specific analysis below, we mainly divide medical institutions into the following categories. According to the level of the institution, medical institutions are divided into secondary medical institutions and tertiary medical institutions. According to the specialty, medical institutions are divided into traditional Chinese medicine (TCM) hospitals, orthopedic hospitals, mental hospitals, tumor hospitals, children’s hospitals, and maternity hospitals.

### 2.2. Modeling Hospital Visits by Gravity Model

#### 2.2.1. Identify Demographic Units

The population data collected were based on the point-like data of residential buildings. To make better use of the population data based on residential buildings, this paper meshes the population data of residential buildings. First, the city was divided into several squares with sides equal to 1000 m, which serve as the basic geographical unit of Shanghai’s population statistics. Subsequent calculations of patient flow were also based on this geographic unit. As a result, Shanghai was divided into 6699 geographical units, including many irregular grids of geographic units close to administrative boundaries. Incomplete geographical units were caused by the irregular regional boundaries of Shanghai, and they would not hinder the subsequent calculations of network distance. Each geographic unit was identified by a unique code (i) that facilitates subsequent calculations.

#### 2.2.2. Establishment of the Patient Distribution Flow Analysis Database

Combining population data, inpatient data, and hospital bed information, a database of the distribution flow matrix of patients was established. The specific way to integrate the three databases is as follows.

(1) Establish the inpatient flow database ($T_{ij}$). First, according to the home address information of inpatients in the inpatient database, the spatial locations of the inpatients were identified. Second, with the help of geographic information systems, the spatial relationship between the patient’s home address and geographic unit was calculated. Then, the number of patients accepted by each medical institution in each geographic unit were summarized and counted.
(2) Count the population in each geographic unit ($P_{i}$). According to the spatial relationship between residential sites and geographical units, the resident population of each geographical unit was calculated.
(3) Calculate the distance matrix between each geographic unit and each hospital ($d_{ij}$). First, each medical institution was given a unique identification code. Second, traffic road information was superimposed, and the road network distance between each geographic unit and medical institution was calculated by using ArcGIS to form the distance matrix of geographical units (i) and medical institutions (j).
(4) Add the population information from step 2 ($P_{i}$) to the inpatient flow database ($T_{ij}$). The unique identifier of the geographic units was used as the connection field to add the geographic unit population information attributed to the inpatient flow database.
(5) Add the distance information from step 3 ($d_{ij}$) to the inpatient flow database ($T_{ij}$). The geographic unit and the hospital unique identifiers were used as connection fields to add the distance attribute between the geographic units and the hospitals to the inpatient flow database.
(6) Add the hospital bed information ($S_{j}$) to the inpatient flow database ($T_{ij}$). The unique hospital identification code was used as a connection field to add attributes, such as the number of beds from the hospital database, to the inpatient flow database.

Based on the above steps, we established the patient flow database, which included key data such as the population, supply, distance, and flow.

#### 2.2.3. Nonlinear Fitting of Inpatient Flow Patterns

The interaction between residents and hospitals, measured by the volume of discharges from hospital j to demographic unit i, denoted by $T_{ij}$, was formulated as a gravity model:

(1) $$T_{ij}=uP_{i}^{\alpha }S_{j}^{\sigma }f\left(d_{ij}\right)$$

where $P_{i}$ was the population in each geographical unit i. $S_{j}$ was the number of beds in hospital j. $d_{ij}$ was the travel distance from geographical unit i to hospital j in kilometers, and $f\left(d_{ij}\right)$ was a generalized distance decay function. μ was a scalar. **α** was the parameter describing the effect of the population numbers in each geographic unit on the interaction. **σ** was the parameter describing the effect of the number of hospital beds on the interaction. How can the distance decay function $f\left(d_{ij}\right)$ be defined? The power [14], negative exponential [10], Gaussian [15], and log-logistic distributions [16] were popular, and can be found for describing the distance decay of hospital utilization in the literature [17]. In the empirical study of the Shanghai inpatient pattern, these four functions were used as distance attenuation functions for fitting. The specific functions were listed in Table 1. The unknown parameters (μ, α, σ, β) in these models can be estimated by nonlinear regression. The adjusted coefficient of determination (R^2^) and Akaike information criterion (AIC) values were used to test the fitting effect of the models [18]. SAS 9.4 was used to fit the models. The adjusted R^2^ was defined as the portion of dependent variable’s variation explained by a nonlinear regression model. The AIC was used to measure the relative quality of the models due to varying complexities of four functions: four parameters when a power, exponential, and Gaussian model was used, and five parameters in the log-logistic model. The model with the maximum adjusted R^2^ and minimum AIC was selected as the best-fitting model.

## 3. Results

In the empirical analysis of the Shanghai inpatient flow pattern, the power function, the negative exponential function, the Gaussian function, and the log-logistic function models were used to simulate and analyze the distribution of patients at different medical institutions of different levels and specialties in Shanghai. The simulation results were shown in Table 2. For all the hospitalized patients, tertiary hospital inpatients, regional hospital inpatients, the traditional Chinese medicine hospital inpatients, orthopedic hospital inpatients, and mental hospital inpatients, the four distance-decay functions were valid, among which the log-logistic model had the best-fitting effect. In contrast, for inpatients at tumor hospitals, children’s hospitals, and maternity hospitals, the power function, the negative exponential function, and the Gaussian function models were feasible, but the model fitting effect was poor; the log-logistic function model was not feasible. In general, from the perspective of model selection, the log-logistic function model had a good simulation effect and the strongest applicability in most hospitals.

**Figure 1:**
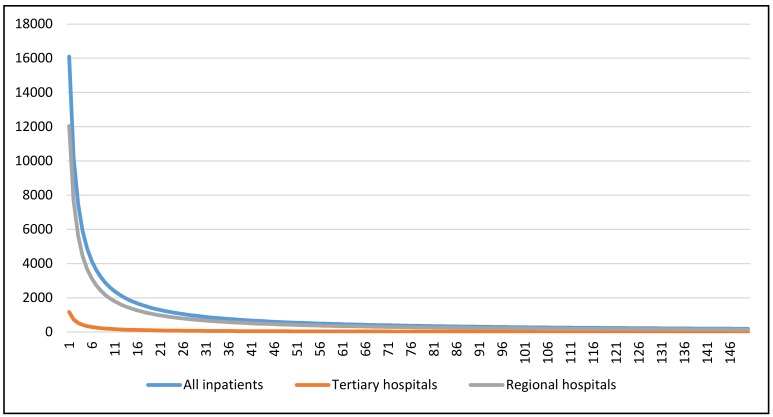
Inpatient distribution pattern among tertiary hospitals and regional hospitals captured by the fitted log-logistic function model.

The results of comparing the distribution pattern of inpatients among different levels of hospitals were as follows. Figure 1 showed the differences in the inpatient distribution patterns among tertiary hospitals and regional hospitals captured by the fitted log-logistic function models. The optimal log-logistic curve for each subgroup was drawn with P_i_ and S_j_ both set to 1000. As the results show, regional hospital patient flow increased rapidly with increasing distance attenuation (β = 1.89), and tertiary hospital patient flow increased significantly more slowly with increasing distance attenuation speed (β = 1.40). The decline rate of patient flow with increasing distance in all hospitals was between the two (β = 1.67).

**Figure 2:**
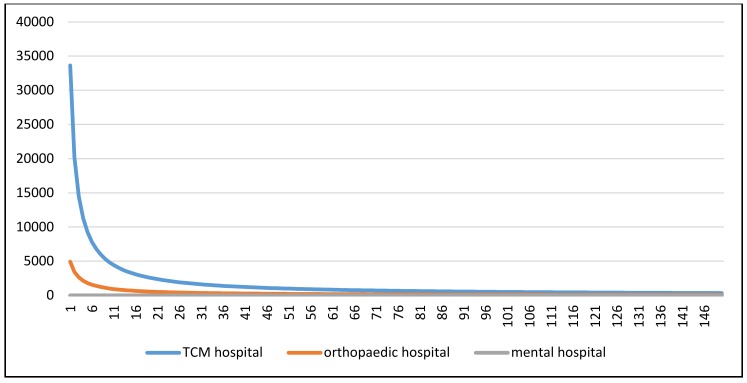
Inpatient distribution pattern among tertiary hospitals and regional hospitals captured by the fitted log-logistic function model.

The results of comparing the distributions of inpatients among different specialties of hospitals are as follows. Figure 2 showed the difference in the inpatient distribution patterns among traditional Chinese medicine hospitals, orthopedic hospitals, and mental hospitals captured by the fitted log-logistic function models. The optimal log-logistic curve for each subgroup was drawn with P_i_ and S_j_ both set to 1000. As the results show, the increase in patient flow at traditional Chinese medicine hospitals with increasing distance attenuation was the fastest (**β** = 1.64), and that at the mental hospital and orthopedic hospital was slower (β = 0.85).

In general, this paper analyzed the differences in the patient distribution model of hospitals of different levels and analyzed the differences in the patient distribution models for different specialized hospitals. Based on these analyses, the model of the inpatient distribution in Shanghai and the values of key parameters were given (Table 3).

## 4. Discussion

In terms of the model selection of patient distribution patterns, the empirical study of Shanghai and previous studies reached similar results, and the log-logistic function model was found to be the best-fitting model for the patient distribution. Some studies have conducted empirical studies on the inpatient distribution in Florida [5] and obtained the fitting results. Compared with the attenuation coefficient of hospitalized patients in Florida, the distance attenuation rate of all hospitalized patients in Shanghai was slower, as the distance attenuation coefficient of all hospitalized patients in Shanghai was 1.67, and that of hospitalized patients in Florida was 2.14. There may be many reasons for the distance attenuation coefficient difference, such as the service area, population density, and concentration of medical resources. All of these factors will result in different distance attenuation coefficients.

By comparing the differences in the distribution patterns of hospitalized patients in regional and tertiary medical institutions in Shanghai, it can be found that the distance attenuation coefficient of hospitalized patients at regional hospitals is larger than that of patients at tertiary hospitals, which means that the distribution attenuation rate of hospitalized patients in regional hospitals is faster. Tertiary hospitals always have more medical resources with better medical quality and advanced medical level, resulting in a larger range of patients, so residents are willing to pay more for medical treatment [19,20,21]. In a previous study, a tertiary hospital in Shanghai was taken as an example, and the attenuation coefficient of inpatients at this hospital was verified by using an exponential function model based on the distribution data of inpatients. The distance attenuation coefficient for this tertiary hospital in Shanghai was 0.7 [7]. The results were similar to the estimated distance attenuation coefficient of inpatients in tertiary hospitals in this paper, which showed that the estimated value of β is 0.95 under the power function model, and β is 1.4 under the log-logistic function model.

By comparing the differences in the distribution patterns of inpatients at specialized hospitals in Shanghai, it can be found that the distribution model of patients in specialized hospitals is more complicated. In this paper, the distribution fitting effect of patients in tumor hospitals, children’s hospitals and maternity hospitals was not good. This phenomenon may be due to the different service objectives and contents of different specialties, resulting in poor comparability between the distribution patterns of patients among the different specialties. At the same time, there were large differences in the technical levels among specialized hospitals, which may further lead to a poor fitting effect of the distribution of patients at specialized hospitals. It should be noted that the inpatient distribution model estimated from the specialist hospitals in this article cannot completely replace the distribution of all specialist inpatients because many inpatients for specific specialties go to general medical institutions instead of specialized hospitals. This kind of inpatient data was not included in the analysis of the inpatient distribution model in specialized hospitals in this paper.

The main advantages of this paper were that it collected the actual inpatient distribution data in Shanghai and demonstrated the distribution pattern of inpatients in Shanghai. At the same time, according to the different levels and specialties of medical institutions, the distribution patterns of inpatients at different levels and different specialized hospitals were explored. This study provided more empirical evidence for patient distribution models. In addition, it provided a research basis for further analyzing scientific issues, such as service area, market scope, and medical resource access at medical institutions [22].

The main disadvantage was that only local Shanghai inpatients were included in the empirical analysis of the Shanghai inpatient distribution pattern. However, Shanghai, as the medical center for eastern China, can reach areas beyond Shanghai. Therefore, this study was limited by including only Shanghai patients to analyze the distribution model of patients at Shanghai medical institutions. However, the inpatient distribution model for regional medical institutions was not affected by this choice because regional hospitals mainly serve local Shanghai residents. In future studies, the scope of the study can be expanded, and the fitting results may be more in line with the distribution characteristics of inpatients in medical institutions in Shanghai.

## 5. Conclusions

This article toke Shanghai as an example to analyze the actual distributions of inpatients, used the power function, the negative exponential function, the Gaussian function, and the log-logistic function model to fit the distribution of inpatients, and derived a model of inpatient distributions in Shanghai based on the four models. Finally, the adjusted R^2^ value and AIC value were used to determine the model with the best-fitting effect. Based on the results of empirical analysis, we believe that the four attenuation coefficient models are valid in the Shanghai empirical study, and the log-logistic function model of the inpatient distributions at most hospitals has a good simulation effect. The model of the inpatient distribution in Shanghai and the values of key parameters were given in Table 3. For all the hospitalized patients in Shanghai, the recommended distance attenuation coefficient value based on the log-logistic function model is 1.67. For inpatients at tertiary hospitals, the recommended distance attenuation coefficient value based on the log-logistic function model is 1.4. For inpatients at regional hospitals, the recommended distance attenuation coefficient value based on the log-logistic function model is 1.89. For inpatients at TCM hospitals, the recommended distance attenuation coefficient value based on the log-logistic function model is 1.64. For inpatients at orthopedic hospitals, the recommended distance attenuation coefficient value based on the log-logistic function model is 0.85. For inpatients at mental hospitals, the recommended distance attenuation coefficient value based on the log-logistic function model is 0.85. As an important parameter in the patient distribution model, distance attenuation coefficient can help researchers intuitively understand the distribution attenuation rate of different types of patients. The recommended distance attenuation coefficient can provide reference values for key parameters of the patient distribution model for extended studies. However, the simulations of the inpatient distribution patterns for the tumor hospitals, children’s hospitals, and maternity hospitals in Shanghai were not good. Therefore, further in-depth analysis, combined with the characteristics of specific specialties, are needed to obtain the inpatient distribution model in line with the characteristics of these specialties.

## Figures and Tables

**Table 1:** Distance-decay functions and the corresponding gravity models.

Distance Decay Functions	Formula $f\left(d_{ij}\right)$	Formula $T_{ij}$
Power function	$d_{ij}^{-\beta }$	$T_{ij}=uP_{i}^{\alpha }S_{j}^{\sigma }d_{ij}^{-\beta }$
Negative exponential function	$e^{-\beta d_{ij}}$	$T_{ij}=uP_{i}^{\alpha }S_{j}^{\sigma }e^{-\beta d_{ij}}$
Gaussian function	$e^{-{\left(\frac{d_{ij}}{\theta }\right)}^{\frac{2}{2}}}$	$T_{ij}=uP_{i}^{\alpha }S_{j}^{\sigma }e^{-{\left(\frac{d_{ij}}{\theta }\right)}^{\frac{2}{2}}}$
Log-logistic function	$1/\left(1+{\left(\frac{d_{ij}}{\theta }\right)}^{\beta }\right)$	$T_{ij}=uP_{i}^{\alpha }S_{j}^{\sigma }/\left(1+{\left(\frac{d_{ij}}{\theta }\right)}^{\beta }\right)$

**Table 2:** Simulation results of the distribution of patients at different medical institutions of different levels and specialties in Shanghai.

Hospital Type	No. Inpatients	Formula f(dij)	Parameters					Model Assessment		
			μ	α	σ	θ	β	P	R^2^	AIC
All inpatients	2524709	Power	0.00116	0.5572	1.0605	-	0.78	<0.0001	16.07	7050593
		Exponential	0.0918	0.4117	0.7899	-	0.4669	<0.0001	20.34	6993253
		Gaussian	0.0469	0.4506	0.7767	2.0439	-	<0.0001	18.41	7019565
		Log-logistic	0.1206	0.3991	0.7901	1.2256	1.6696	<0.0001	21.61	6975704
Tertiary hospitals	966405	Power	0.000152	0.9266	0.828	-	0.9452	<0.0001	19.31	2795705
		Exponential	0.00374	0.7035	0.7766	-	0.3933	<0.0001	18.81	2798303
		Gaussian	0.00141	0.7412	0.7935	2.6672	-	<0.0001	16.71	2808981
		Log-logistic	0.0055	0.7250	0.7602	0.888	1.4046	<0.0001	20.21	2790959
Regional hospitals	1558304	Power	0.0104	0.2940	1.1436	-	0.7456	<0.0001	15.77	4209599
		Exponential	0.0674	0.3758	0.9265	-	0.5245	<0.0001	22.82	4150433
		Gaussian	0.0299	0.4257	0.9251	1.7796	-	<0.0001	21.37	4163078
		Log-logistic	0.0874	0.3483	0.9222	1.4221	1.8920	<0.0001	23.97	4140329
TCM hospital	313983	Power	0.00391	0.4808	1.0125		0.7580	<0.0001	41.50	726665
		Exponential	0.2610	0.3481	0.8133	0.7009		<0.0001	52.97	696918
		Gaussian	0.2464	0.3446	0.7837	1.2839		<0.0001	48.73	708687
		Log-logistic	0.3884	0.3093	0.8311	0.8315	1.6358	<0.0001	57.35	745253
Orthopedic hospital	1715	Power	0.1647	0.3108	0.2		0.4234	<0.0001	22.53	1983
		Exponential	0.0113	0.6089	0.1791		0.1229	<0.0001	21.9	1989
		Gaussian	0.00963	0.5427	0.2617	9.3228		<0.0001	19.74	2009
		Log-logistic	0.2284	0.4228	0.0903	1.1018	0.848	<0.0001	23.82	1970
Mental hospital	9209	Power	0.000003054	0.9505	0.8436		0.3335	<0.0001	38.56	8473
		Exponential	0.000009653	0.8663	0.8047		0.0729	<0.0001	39.29	8425
		Gaussian	0.000004937	0.9082	0.7909	12.341		<0.0001	37.64	8532
		Log-logistic	0.000015	0.8653	0.8209	3.0989	0.8531	<0.0001	40.19	8365
Tumor hospital	23486	Power	0.00373	0.5441	0.6464	-	0.4581	<0.0001	2.58	67072
		Exponential	0.1074	0.1602	0.7724	-	0.1290	<0.0001	2.82	67047
		Gaussian	0.0774	0.0715	0.9204	6.8460		<0.0001	2.86	67043
		Log-logistic								
Children’s Hospital	55000	Power	2.3227	0.3625	0.0575		0.7137	<0.0001	0.83	192687
		Exponential	5.5942	0.2269	0.1188		0.1339	<0.0001	0.98	192651
		Gaussian	4.1994	0.2065	0.1516	7.1108		<0.0001	1.04	192639
		Log-logistic								
Maternity hospital	37976	Power	0.000658	−0.3234	2.9667		1.3515	<0.0001	2.43	152093
		Exponential	0.000485	−0.5219	3.6051		0.7271	<0.0001	5.98	151481
		Gaussian	2.15E-11	−3.1238	11.412	0.7316		<0.0001	13.81	150048
		Log-logistic								

**Table 3:** Estimated distance attenuation coefficient β and the inpatient distribution model for Shanghai.

Hospital Type	β	T_ij_
All	1.67	$T_{ij}=0.12P_{i}^{0.4}S_{j}^{0.8}/\left(1+{\left(\frac{d_{ij}}{1.23}\right)}^{1.67}\right)$
Tertiary hospitals	1.40	$T_{ij}=0.01P_{i}^{0.73}S_{j}^{0.76}/\left(1+{\left(\frac{d_{ij}}{0.89}\right)}^{1.4}\right)$
Regional hospitals	1.89	$T_{ij}=0.09P_{i}^{0.35}S_{j}^{0.92}/\left(1+{\left(\frac{d_{ij}}{1.42}\right)}^{1.89}\right)$
TCM hospital	1.64	$T_{ij}=0.39P_{i}^{0.31}S_{j}^{0.83}/\left(1+{\left(\frac{d_{ij}}{0.83}\right)}^{1.64}\right)$
Orthopedic hospital	0.85	$T_{ij}=0.23P_{i}^{0.42}S_{j}^{0.09}/\left(1+{\left(\frac{d_{ij}}{1.1}\right)}^{0.85}\right)$
Mental hospital	0.85	$T_{ij}=0.000015P_{i}^{0.87}S_{j}^{0.82}/\left(1+{\left(\frac{d_{ij}}{3.10}\right)}^{0.85}\right)$
